# Synthesis, crystal structure and catalytic activity in reductive amination of di­chlorido­(η^6^-*p*-cymene)(2′-di­cyclo­hexyl­phosphanyl-2,6-di­meth­oxy­biphen­yl-κ*P*)ruthenium(II)

**DOI:** 10.1107/S2056989018003821

**Published:** 2018-03-09

**Authors:** Maria Makarova, Alexey A. Tsygankov, Olga Chusova, Ivan V. Linko, Pavel V. Dorovatovskii, Yan V. Zubavichus

**Affiliations:** a Mendeleev University of Chemical Technology of Russia, Miusskaya sq. 9, Moscow 125047, Russian Federation; b Research Institute of Chemistry, Peoples’ Friendship University of Russia (RUDN University), 6 Miklukho-Maklay St., Moscow 117198, Russian Federation; c Inorganic Chemistry Department, Peoples’ Friendship University of Russia (RUDN University), 6 Miklukho-Maklay St., Moscow 117198, Russian Federation; d National Research Centre Kurchatov Institute, 1 Acad. Kurchatov Sq., Moscow 123182, Russian Federation

**Keywords:** crystal structure, ruthenium(II), η^6^-arene complex, phosphine complex, reductive amination, carbon monoxide, synchrotron radiation, hydrogen bonding, C—H⋯π inter­actions

## Abstract

The title compound, [RuCl_2_(C_10_H_14_)(C_26_H_35_O_2_P)] (**I**), crystallizes in the monoclinic space group *P*2_1_/*c* with two crystallographically independent mol­ecules (*A* and *B*) in the asymmetric unit. The geometries of both mol­ecules are very similar and distinguished only by the twist angles of the two benzene rings in the phosphine substituents [89.54 (14) and 78.36 (14)° for mol­ecules *A* and *B*, respectively]. The Ru atoms have classical pseudo-tetra­hedral piano-stool coordination environments. The conformation of each mol­ecule is stabilized by intra­molecular C—H⋯O and C—H⋯Cl hydrogen bonds and C—H⋯π inter­actions. The two mol­ecules are linked by a C—H⋯Cl hydrogen bond. In the crystal, the mol­ecules are further linked by C—H⋯ π inter­actions, forming –*A*–*B*–*A*–*B*– chains propagating along the *a*-axis direction. Complex **I** is an active catalyst for reductive amination reaction. The catalytic activity of this complex can be explained by the lability of the *p*-cymene ligand, which can be replaced by two-electron ligands such as CO or amine.

## Abstract 2

The synthesis, crystal structure and catalytic activity in reductive amination reactions of a new ruthenium complex are described.

## Chemical context   

The design of new organometallic complexes is important for the development of new catalytic processes as well as for understanding those already known. Recently, a new method­ology for reductive amination in the presence of carbon monoxide as the reducing agent, catalysed by rhodium (Chusov & List, 2014 ▸; Afanasyev *et al.*, 2016 ▸; Yagafarov *et al.*, 2015 ▸), iridium (Moskovets *et al.*, 2017 ▸; Molotkov *et al.*, 2017 ▸) and ruthenium (Kolesnikov *et al.*, 2015 ▸; Afanasyev *et al.*, 2017 ▸) has been described. This protocol is based on the de­oxy­genation potential of CO and does not require an external hydrogen source. This methodology is therefore potentially more selective for those substrates bearing groups that are sensitive to hydrogenation. As a result of the high cost of rhodium and iridium, the development of new catalytic systems based on more abundant metals is important. It has previously been shown that addition of phosphines to ruthenium systems, which were supposed to stabilize catalytic species, dramatically decreases the activity of the catalytic system. To further understand this process and the role of phosphines, the title complex, **I**, was synthesized and its crystal structure and catalytic properties are reported herein.
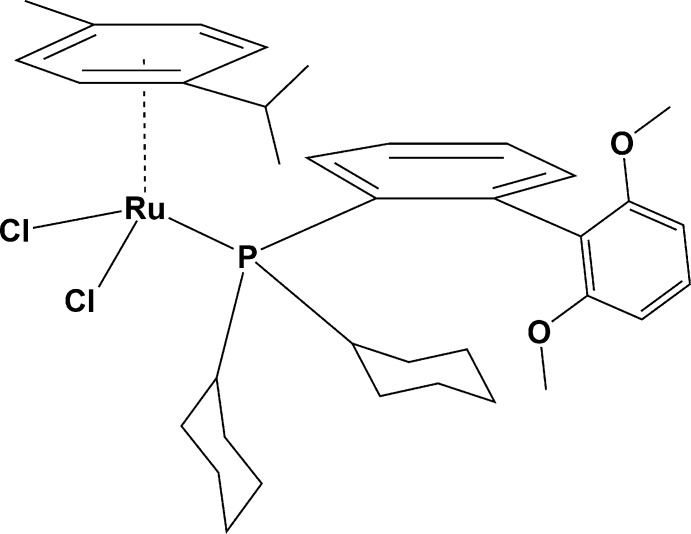


Such η^6^-arene Ru^II^ complexes with piano-stool coordination are known to be active catalysts in different processes (Therrien, 2009 ▸), including hydrogenation (Moldes *et al.*, 1998 ▸), hydro­boration (Kaithal *et al.*, 2016 ▸), transfer hydrogenation (Aznar *et al.*, 2013 ▸; Cerón-Camacho *et al.*, 2006 ▸; Clavero *et al.*, 2016 ▸) and isomerization of allylic alcohols (Díaz-Álvarez *et al.*, 2006 ▸; Baraut *et al.*, 2015 ▸). Moreover, such complexes have shown promising medicinal properties (Naza­rov *et al.*, 2014 ▸), including anti­cancer activity (Chuklin *et al.*, 2017 ▸).

## Structural commentary   

**Figure 1:**
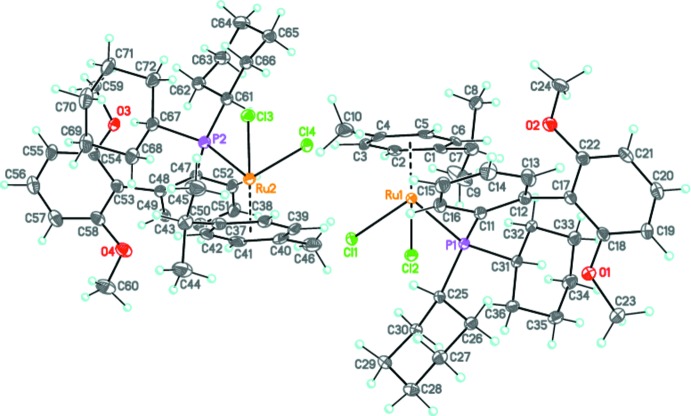
A view of the mol­ecular structure of compound **I**, with atom labelling. Displacement ellipsoids are shown at the 50% probability level.

**Figure 2:**
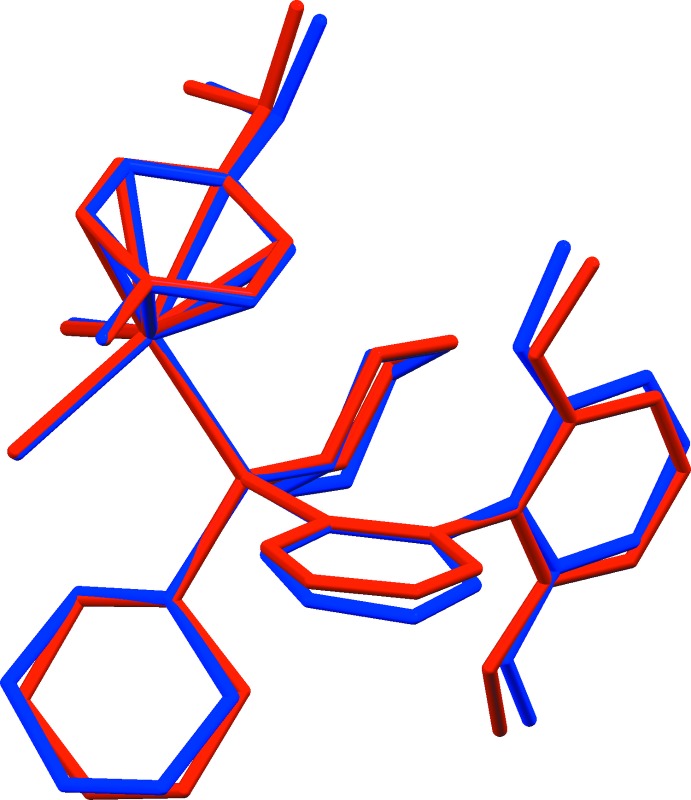
A view of the mol­ecular overlap of the inverted mol­ecule *B* (red) on mol­ecule *A* (blue). H atoms have been omitted for clarity.

The title compound, **I**, crystallizes in the monoclinic space group *P*2_1_/*c* with two crystallographically independent mol­ecules (*A* and *B*, comprising Ru1 and Ru2, respectively) in the asymmetric unit (Fig. 1 ▸). The geometries of both mol­ecules are very similar, as illustrated in Fig. 2 ▸, showing the mol­ecular overlap of the inverted mol­ecule *B* on mol­ecule *A* [r.m.s. deviation of 0.227 Å; *Mercury* (Macrae *et al.*, 2008 ▸)]. They are distinguished only by the twist angles of the two benzene rings in the phosphine substituents [89.54 (14)° for A and 78.36 (14)° for *B*].

**Figure 3:**
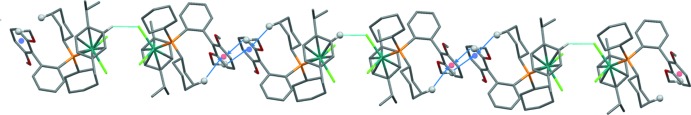
A view of the C—H⋯Cl hydrogen bonds (dashed lines) and the C—H⋯π inter­actions (blue arrows) leading to the formation of chains along [100]; see Table 1 ▸ for details. Only the H atoms involved in these inter­actions are shown, and the centroid in mol­ecule *A* is red, while the centroid in mol­ecule *B* is blue.

The ruthenium atom in each mol­ecule has a classical pseudo-tetra­hedral piano-stool coordination environment, being ligated by two chlorides, the phosphine 2-di­cyclo­hexyl­phosphino-2,6′-di­meth­oxy­biphenyl (SPhos) and an η^6^-*p-*cymene ligand. Owing to steric hinderance, the average Ru—C [2.248 (3) Å], Ru—P [2.4194 (11) Å] and Ru—Cl [2.4455 (11) Å] bond lengths are slightly elongated in comparison with those observed previously in related ruthenium complexes (Muller & Davis, 2012 ▸; Granville *et al.*, 2012 ▸). The bond angles Cl—Ru—Cl [88.04 (4)° for *A* and 86.26 (4)° for *B*] and Cl—Ru—P [87.23 (4) and 87.53 (4) for *A* and 87.86 (4), 87.28 (4)° for *B*] fall within the normal range for known analogous complexes. The meth­oxy groups are coplanar to the parent benzene rings (r.m.s. deviations are 0.070 Å for *A* and 0.082 Å for *B*). In each mol­ecule there are intra­molecular C—H⋯O and C—H⋯Cl hydrogen bonds and C—H⋯π contacts present (see Table 1 ▸), and the two mol­ecules are linked by the C38–H38⋯Cl1 hydrogen bond (Table 1 ▸ and Fig. 3 ▸).

## Supra­molecular features   

**Figure 4:**
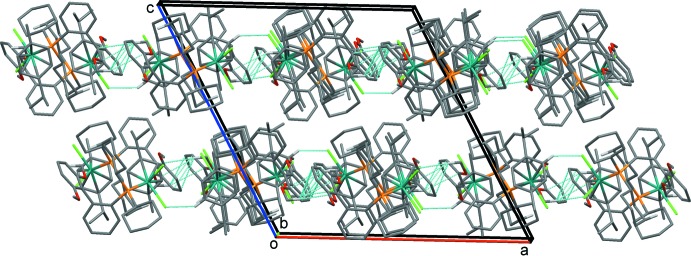
A view along the *b* axis of the crystal packing of compound **I**. The inter­molecular inter­actions are shown as dashed lines (see Table 1 ▸), and only those H atoms involved in these inter­actions have been included.

In the crystal of **I**, mol­ecules are linked by a C—H⋯Cl hydrogen bond and C—H⋯π inter­actions forming –*A*–*B*–*A*–*B*– chains propagating along [100]; details are shown in Fig. 3 ▸ and Table 1 ▸. The overall packing in the crystal structure of **I** is illustrated in Fig. 4 ▸. There are no other significant inter­molecular inter­actions present in the crystal structure.

## Catalytic activity   

**Figure 5:**
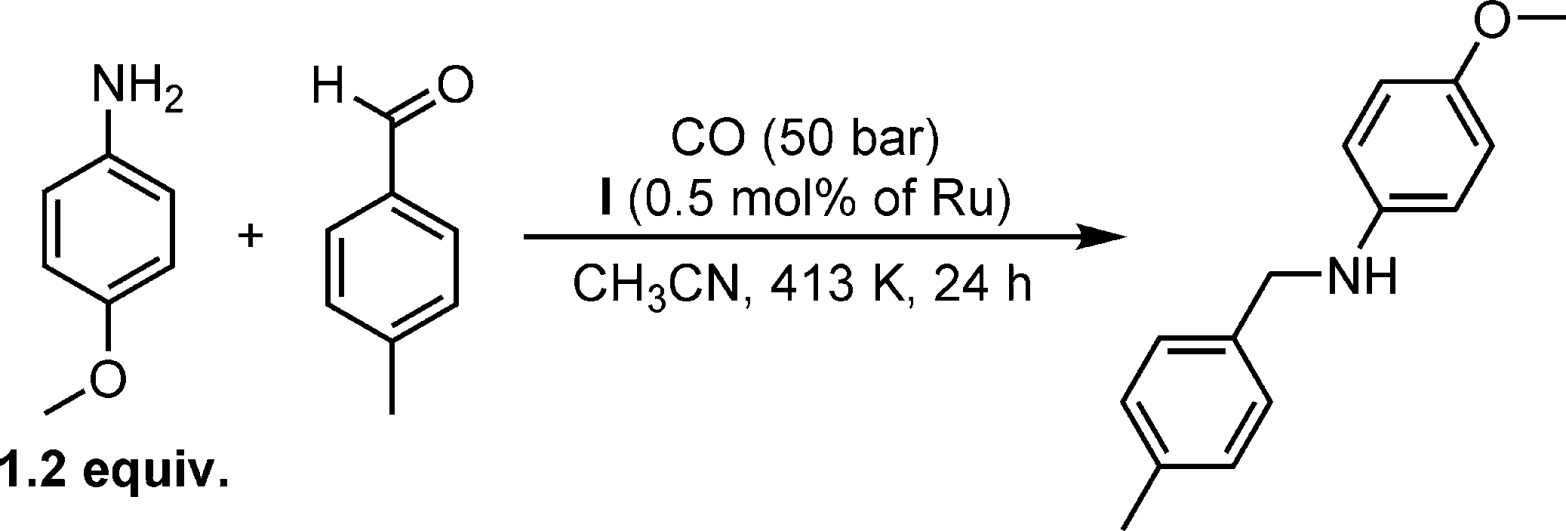
A model reductive amination reaction between *p-*tolu­aldehyde and *p-*anisidine catalyzed by complex **I**.

The catalytic activity was investigated in a model reductive amination reaction between *p-*tolu­aldehyde and *p-*anisidine in conditions similar to those reported previously for ruthenium systems (Fig. 5 ▸). We were delighted to find out that complex **I** was active and furnished the desired amine in 61% yield. The catalytic activity of this complex can be explained by the lability of the *p*-cymene ligand, which can be replaced by two-electron ligands such as CO or amine. The role of the phosphine ligand is in the stabilization of catalytically active species [RuCl_2_SPhos*L_x_*]. Inter­estingly, the dimeric precursor of **I** – [Ru(*p-*cymene)Cl]_2_Cl_2_ – was two times less active (the amine yield is 34%), which can be explained by dissociation of the *p*-cymene ligands followed by aggregation of non-stabil­ized RhCl species. In summary, complex **I** is an active catalyst for reductive amination, and further tuning of phosphine ligands may result in even more active complexes.

## Procedure for reductive amination   

A glass vial in a 10 ml stainless steel autoclave was charged with 0.5 mol% of the catalyst, CH_3_CN, 1.2 equiv. of the *p*-anisidine and 1 equiv. of the *p*-tolualdehyde (the use of a glass vial is crucial: inter­action of the catalyst with the metal surface inside the autoclave can lead to decreased catalytic activity). The autoclave was sealed, flushed three times with 5 bar of carbon monoxide (CO), and then charged with 50 bar of CO. The reactor was placed in an oil bath preheated to 413 K. After the indicated time, the reactor was cooled to room temperature and depressurized. The residue was purified by flash chromatography on silica gel using di­chloro­methane as eluent. ^1^H NMR [400 MHz, CDCl_3_, δ (ppm), *J* (Hz)]: 7.27 (*d*, *J* = 8.1, 2H), 7.16 (*d*, *J* = 8.1, 2H), 6.79 (*d*, *J* = 8.8, 2H), 6.61 (*d*, *J* = 8.8, 2H), 4.25 (*s*, 2H), 3.75 (*s*, 3H), 2.36 (*s*, 3H).

## Synthesis and crystallization   

To a di­chloro­methane (7 ml) solution of [(*p*-cymene)RuCl_2_]_2_ (0.050 g, 0.082 mmol) was added SPhos (69 mg, 0.168 mmol). The dark-orange solution was stirred at room temperature for 24 h. The mixture was partially evaporated under reduced pressure, and the complex precipitated with diethyl ether (10 ml) to give a dark-orange solid (37 mg, 66%). Dark-orange prismatic crystals were obtained by slow diffusion of pentane into the di­chloro­methane solution of complex **I**.

Spectroscopic data: ^1^H NMR [CDCl_3_, 600 MHz, 230 K, δ (ppm), *J* (Hz)]: 8.24 (*dd*, *J* = 13.1, 7.7, 1H), 7.43–7.31 (*m*, 3H), 6.86 (*d*, *J* = 7.2, 1H), 6.71 (*dd*, *J* = 23.2, 7.2, 2H), 5.46 (*d*, *J* = 6.2, 1H), 5.32 (*d*, *J* = 6.2, 1H), 5.16 (*d*, *J* = 6.1, 1H), 5.05 (*d*, *J* = 5.8, 1H), 3.82 (*s*, 3H), 3.73 (*s*, 3H), 2.72–2.52 (*m*, 3H), 2.40 (*q*, *J* = 12.9, 1H), 1.93 (*d*, *J* = 12.1, 1H), 1.73 (*q*, *J* = 12.6, 1H), 1.61 (*s*, 4H), 1.65–1.43 (*m*, 6H), 1.39–1.26 (*m*, 4H), 1.18 (*dd*, *J* = 17.5, 6.8, 6H), 1.26–1.14 (*m*, 1H), 1.08–0.92 (*m*, 3H), 0.42 (*q*, *J* = 13.2, 1H), 0.10 (*q*, *J* = 13.2, 1H). ^13^C NMR [CDCl_3_, 151 MHz, 230 K, δ (ppm), *J* (Hz)]: 158.3, 157.5, 139.6 (*d*, *J* = 27.6), 136.3, 135.9 (*d*, *J* = 15.1), 132.5 (*d*, *J* = 6.3), 130.0, 128.9, 127.6 (*d*, *J* = 10.5), 119.9, 110.5, 104.1, 104.0, 95.4, 88.9, 88.4, 86.9 (*d*, *J* = 6.4), 80.4 (*d*, *J* = 10.1), 55.8, 55.7, 41.4 (*d*, *J* = 18.3), 35.9 (*d*, *J* = 18.4), 33.4, 30.8, 30.0, 29.3, 28.6 (*d*, *J* = 7.6), 28.0 (*d*, *J* = 8.9), 27.6 (*d*, *J* = 14.5), 27.5, 26.8 (*d*, *J* = 14.2), 26.6, 25.4, 22.6, 22.4, 17.3. ^31^P NMR [CDCl_3_, 121 MHz, 220 K, δ (ppm)]: 39.06.

## Refinement   

Crystal data, data collection and structure refinement details are summarized in Table 2 ▸. The hydrogen atoms were placed in calculated positions and refined using a riding model: C—H = 0.95–1.00 Å with *U*_iso_(H) = 1.5*U*_eq_(C-meth­yl) and 1.2U_eq_(C) for other H atoms.

The X-ray diffraction study was carried out on the ‘Belok’ beamline of the National Research Center Kurchatov Institute (Moscow, Russian Federation) using a Rayonix SX165 CCD detector.

A rather large number of reflections (*ca* 100) were omitted in the final cycles of refinement for the following reasons:

(1) In order to achieve better *I*/σ statistics for high-angle reflections we selected exposure times to allow a small fraction of intensity overloads in the low-angle part of the detector. These low-angle reflections with imprecisely measured intensities were excluded from the final cycles of refinement.

2) In the present setup of the synchrotron diffractometer, the low-temperature device eclipses a small region of the image-plate detector near the high-angle limit. This small shadowed region was not masked during integration of the diffraction frames, which erroneously resulted in zero intensity of some reflections.

3) The quality of the single crystal chosen for the diffraction experiment was not perfect. Some systematic intensity distortions may be due to extinction and defects present in the crystal.

## Supplementary Material

Crystal structure: contains datablock(s) global, I. DOI: 10.1107/S2056989018003821/su5428sup1.cif

Structure factors: contains datablock(s) I. DOI: 10.1107/S2056989018003821/su5428Isup2.hkl

CCDC reference: 1827568

Additional supporting information:  crystallographic information; 3D view; checkCIF report

## Figures and Tables

**Table 1:** Hydrogen-bond geometry (Å, °) *Cg*3 and *Cg*8 are the centroids of rings C17–C22 and C53–C58, respectively.

*D*—H⋯*A*	*D*—H	H⋯*A*	*D*⋯*A*	*D*—H⋯*A*
C6—H6⋯O2	0.95	2.50	3.328 (6)	146
C30—H30*A*⋯Cl2	0.99	2.74	3.552 (4)	139
C45—H45*C*⋯Cl3	0.98	2.79	3.577 (5)	137
C46—H46*C*⋯Cl4	0.98	2.68	3.302 (4)	122
C62—H62*A*⋯O3	0.99	2.58	3.261 (6)	126
C66—H66*B*⋯Cl4	0.99	2.71	3.388 (4)	126
C72—H72*A*⋯Cl3	0.99	2.78	3.617 (6)	143
C38—H38⋯Cl1	0.95	2.70	3.376 (4)	129
C33—H33*B*⋯*Cg*3	0.99	2.97	3.703 (5)	132
C69—H69*A*⋯*Cg*8	0.99	2.91	3.649 (6)	132
C60—H60*B*⋯*Cg*3^i^	0.98	2.84	3.655 (6)	142
C24—H24*B*⋯*Cg*8^ii^	0.98	2.85	3.710 (6)	147

Symmetry codes: (i) 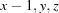; (ii) 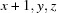.

**Table 2:** Experimental details

Crystal data	
Chemical formula	[RuCl_2_(C_10_H_14_)(C_26_H_35_O_2_P)]
*M*_r_	716.69
Crystal system, space group	Monoclinic, *P*2_1_/*c*
Temperature (K)	100
*a*, *b*, *c* (Å)	19.790 (4), 19.950 (4), 20.225 (4)
β (°)	118.17 (3)
*V* (Å^3^)	7039 (3)
*Z*	8
Radiation type	Synchrotron, λ = 0.96260 Å
μ (mm^−1^)	1.52
Crystal size (mm)	0.20 × 0.15 × 0.10

Data collection	
Diffractometer	Rayonix SX165 CCD
Absorption correction	Multi-scan (*SCALA*; Evans, 2006 ▸)
*T*_min_, *T*_max_	0.730, 0.850
No. of measured, independent and observed [*I* > 2σ(*I*)] reflections	85347, 15331, 11898
*R*_int_	0.080
(sin θ/λ)_max_ (Å^−1^)	0.646

Refinement	
*R*[*F* ^2^ > 2σ(*F* ^2^)], *wR*(*F* ^2^), *S*	0.059, 0.160, 1.07
No. of reflections	15331
No. of parameters	768
H-atom treatment	H-atom parameters constrained
Δρ_max_, Δρ_min_ (e Å^−3^)	1.92, −1.77

Computer programs: *MARCCD* (Doyle, 2011 ▸), *iMosflm* (Battye *et al.*, 2011 ▸), *SHELXT* (Sheldrick, 2015*a* ▸), *SHELXL2014* (Sheldrick, 2015*b* ▸), *SHELXTL* (Sheldrick, 2008 ▸), *Mercury* (Macrae *et al.*, 2008 ▸), *PLATON* (Spek, 2009 ▸) and *publCIF*(Westrip, 2010 ▸).

## supplementary crystallographic information

### Crystal data

**Table:**

[RuCl_2_(C_10_H_14_)(C_26_H_35_O_2_P)]	*F*(000) = 2992
*M**_r_* = 716.69	*D*_x_ = 1.353 Mg m^−^^3^
Monoclinic, *P*2_1_/*c*	Synchrotron radiation, λ = 0.96260 Å
*a* = 19.790 (4) Å	Cell parameters from 600 reflections
*b* = 19.950 (4) Å	θ = 3.2–35.0°
*c* = 20.225 (4) Å	µ = 1.52 mm^−^^1^
β = 118.17 (3)°	*T* = 100 K
*V* = 7039 (3) Å^3^	Prism, dark-orange
*Z* = 8	0.20 × 0.15 × 0.10 mm

### Data collection

**Table:**

Rayonix SX165 CCD diffractometer	11898 reflections with *I* > 2σ(*I*)
φ scan	*R*_int_ = 0.080
Absorption correction: multi-scan (SCALA; Evans, 2006)	θ_max_ = 38.4°, θ_min_ = 3.2°
*T*_min_ = 0.730, *T*_max_ = 0.850	*h* = −25→23
85347 measured reflections	*k* = −25→25
15331 independent reflections	*l* = −26→24

### Refinement

**Table:**

Refinement on *F*^2^	Secondary atom site location: difference Fourier map
Least-squares matrix: full	Hydrogen site location: inferred from neighbouring sites
*R*[*F*^2^ > 2σ(*F*^2^)] = 0.059	H-atom parameters constrained
*wR*(*F*^2^) = 0.160	*w* = 1/[σ^2^(*F*_o_^2^) + (0.0738*P*)^2^ + 8.8*P*] where *P* = (*F*_o_^2^ + 2*F*_c_^2^)/3
*S* = 1.07	(Δ/σ)_max_ = 0.001
15331 reflections	Δρ_max_ = 1.92 e Å^−^^3^
768 parameters	Δρ_min_ = −1.77 e Å^−^^3^
0 restraints	Extinction correction: SHELXL2014 (Sheldrick, 2015b), Fc^*^=kFc[1+0.001xFc^2^λ^3^/sin(2θ)]^-1/4^
Primary atom site location: structure-invariant direct methods	Extinction coefficient: 0.0012 (1)

### Special details

**Table:**

Geometry. All esds (except the esd in the dihedral angle between two l.s. planes) are estimated using the full covariance matrix. The cell esds are taken into account individually in the estimation of esds in distances, angles and torsion angles; correlations between esds in cell parameters are only used when they are defined by crystal symmetry. An approximate (isotropic) treatment of cell esds is used for estimating esds involving l.s. planes.

### Fractional atomic coordinates and isotropic or equivalent isotropic displacement parameters (Å^2^)

**Table:**

	*x*	*y*	*z*	*U*_iso_*/*U*_eq_	
Ru1	0.39465 (2)	0.34941 (2)	0.26968 (2)	0.02059 (11)	
Cl1	0.37597 (5)	0.31613 (4)	0.37623 (5)	0.0248 (2)	
Cl2	0.30004 (5)	0.26463 (4)	0.19505 (5)	0.0264 (2)	
P1	0.49089 (5)	0.26228 (4)	0.30732 (5)	0.0197 (2)	
O1	0.70112 (16)	0.17532 (12)	0.34952 (16)	0.0293 (6)	
O2	0.63517 (16)	0.39635 (13)	0.25883 (17)	0.0329 (6)	
C1	0.3772 (2)	0.41199 (16)	0.1694 (2)	0.0237 (8)	
C2	0.3194 (2)	0.42974 (17)	0.1905 (2)	0.0275 (9)	
H2	0.2668	0.4275	0.1540	0.033*	
C3	0.3389 (3)	0.45023 (17)	0.2638 (3)	0.0325 (10)	
H3	0.2996	0.4630	0.2756	0.039*	
C4	0.4174 (3)	0.45222 (16)	0.3212 (2)	0.0322 (10)	
C5	0.4762 (2)	0.43513 (17)	0.3016 (2)	0.0266 (8)	
H5	0.5287	0.4375	0.3382	0.032*	
C6	0.4548 (2)	0.41436 (16)	0.2261 (2)	0.0255 (8)	
H6	0.4939	0.4018	0.2139	0.031*	
C7	0.3581 (3)	0.39964 (19)	0.0878 (2)	0.0339 (9)	
H7	0.4004	0.3720	0.0882	0.041*	
C8	0.3595 (3)	0.46815 (19)	0.0540 (2)	0.0371 (10)	
H8A	0.3200	0.4971	0.0549	0.056*	
H8B	0.3495	0.4622	0.0021	0.056*	
H8C	0.4099	0.4889	0.0834	0.056*	
C9	0.2837 (4)	0.3638 (3)	0.0395 (3)	0.075 (2)	
H9A	0.2857	0.3185	0.0590	0.113*	
H9B	0.2757	0.3611	−0.0121	0.113*	
H9C	0.2412	0.3886	0.0401	0.113*	
C10	0.4381 (3)	0.4735 (2)	0.4003 (3)	0.0489 (13)	
H10A	0.4028	0.4525	0.4154	0.073*	
H10B	0.4342	0.5224	0.4021	0.073*	
H10C	0.4907	0.4595	0.4345	0.073*	
C11	0.5865 (2)	0.30029 (17)	0.3731 (2)	0.0235 (8)	
C12	0.6558 (2)	0.30373 (18)	0.3684 (2)	0.0255 (8)	
C13	0.7216 (2)	0.3298 (2)	0.4312 (2)	0.0331 (9)	
H13	0.7687	0.3309	0.4294	0.040*	
C14	0.7204 (3)	0.3538 (2)	0.4953 (3)	0.0359 (10)	
H14	0.7659	0.3702	0.5365	0.043*	
C15	0.6510 (2)	0.35352 (19)	0.4982 (2)	0.0315 (9)	
H15	0.6487	0.3708	0.5409	0.038*	
C16	0.5856 (2)	0.32755 (18)	0.4379 (2)	0.0269 (8)	
H16	0.5387	0.3280	0.4401	0.032*	
C17	0.6690 (2)	0.28524 (18)	0.3027 (2)	0.0249 (8)	
C18	0.6972 (2)	0.22167 (18)	0.2973 (2)	0.0258 (8)	
C19	0.7185 (2)	0.2073 (2)	0.2411 (2)	0.0316 (9)	
H19	0.7372	0.1642	0.2380	0.038*	
C20	0.7113 (3)	0.2582 (2)	0.1901 (3)	0.0359 (10)	
H20	0.7253	0.2491	0.1521	0.043*	
C21	0.6841 (2)	0.3218 (2)	0.1940 (2)	0.0335 (9)	
H21	0.6800	0.3557	0.1594	0.040*	
C22	0.6627 (2)	0.33488 (19)	0.2500 (2)	0.0279 (8)	
C23	0.7143 (3)	0.10637 (19)	0.3367 (3)	0.0392 (11)	
H23A	0.6770	0.0932	0.2856	0.059*	
H23B	0.7086	0.0774	0.3728	0.059*	
H23C	0.7663	0.1018	0.3429	0.059*	
C24	0.6260 (3)	0.4486 (2)	0.2060 (3)	0.0403 (11)	
H24A	0.5901	0.4337	0.1551	0.060*	
H24B	0.6757	0.4586	0.2087	0.060*	
H24C	0.6059	0.4891	0.2181	0.060*	
C25	0.4883 (2)	0.19855 (17)	0.3755 (2)	0.0226 (8)	
H25	0.4913	0.2267	0.4177	0.027*	
C26	0.5590 (2)	0.15215 (17)	0.4139 (2)	0.0257 (8)	
H26A	0.5564	0.1164	0.3789	0.031*	
H26B	0.6066	0.1782	0.4288	0.031*	
C27	0.5592 (2)	0.12113 (19)	0.4837 (2)	0.0310 (9)	
H27A	0.5674	0.1570	0.5207	0.037*	
H27B	0.6022	0.0890	0.5072	0.037*	
C28	0.4830 (2)	0.08444 (19)	0.4640 (2)	0.0325 (9)	
H28A	0.4794	0.0439	0.4343	0.039*	
H28B	0.4835	0.0698	0.5110	0.039*	
C29	0.4117 (2)	0.12857 (19)	0.4191 (2)	0.0309 (9)	
H29A	0.3649	0.1012	0.4032	0.037*	
H29B	0.4105	0.1652	0.4516	0.037*	
C30	0.4126 (2)	0.15879 (17)	0.3496 (2)	0.0248 (8)	
H30A	0.3681	0.1890	0.3232	0.030*	
H30B	0.4092	0.1226	0.3146	0.030*	
C31	0.5014 (2)	0.21346 (17)	0.2328 (2)	0.0235 (8)	
H31	0.5564	0.1999	0.2545	0.028*	
C32	0.4824 (2)	0.25709 (17)	0.1633 (2)	0.0253 (8)	
H32A	0.5147	0.2979	0.1791	0.030*	
H32B	0.4281	0.2713	0.1407	0.030*	
C33	0.4959 (3)	0.2195 (2)	0.1038 (2)	0.0327 (9)	
H33A	0.4813	0.2487	0.0596	0.039*	
H33B	0.5510	0.2085	0.1248	0.039*	
C34	0.4483 (3)	0.15464 (19)	0.0796 (2)	0.0358 (10)	
H34A	0.3931	0.1659	0.0533	0.043*	
H34B	0.4606	0.1294	0.0444	0.043*	
C35	0.4661 (3)	0.11097 (19)	0.1486 (2)	0.0325 (9)	
H35A	0.5202	0.0962	0.1715	0.039*	
H35B	0.4334	0.0704	0.1322	0.039*	
C36	0.4525 (2)	0.14818 (17)	0.2079 (2)	0.0264 (8)	
H36A	0.4666	0.1188	0.2519	0.032*	
H36B	0.3975	0.1596	0.1868	0.032*	
Ru2	0.11771 (2)	0.45313 (2)	0.25706 (2)	0.02841 (11)	
Cl3	0.20723 (6)	0.54512 (4)	0.31822 (6)	0.0308 (2)	
Cl4	0.12802 (6)	0.48211 (5)	0.14440 (6)	0.0320 (2)	
P2	0.01492 (6)	0.53415 (5)	0.21645 (6)	0.0281 (2)	
O3	−0.19526 (16)	0.61509 (13)	0.16977 (16)	0.0319 (6)	
O4	−0.12390 (17)	0.40226 (15)	0.28418 (18)	0.0409 (7)	
C37	0.1490 (3)	0.39587 (19)	0.3650 (3)	0.0339 (10)	
C38	0.2032 (2)	0.37920 (18)	0.3386 (2)	0.0293 (9)	
H38	0.2564	0.3846	0.3716	0.035*	
C39	0.1800 (3)	0.35547 (18)	0.2664 (3)	0.0356 (10)	
H39	0.2175	0.3443	0.2515	0.043*	
C40	0.0995 (3)	0.34759 (19)	0.2137 (3)	0.0403 (11)	
C41	0.0448 (3)	0.3627 (2)	0.2383 (3)	0.0414 (11)	
H41	−0.0083	0.3568	0.2053	0.050*	
C42	0.0702 (3)	0.38710 (19)	0.3141 (3)	0.0375 (11)	
H42	0.0332	0.3974	0.3298	0.045*	
C43	0.1769 (3)	0.4108 (2)	0.4482 (3)	0.0438 (12)	
H43	0.1349	0.4347	0.4528	0.053*	
C44	0.1908 (4)	0.3431 (2)	0.4895 (3)	0.0590 (15)	
H44A	0.1430	0.3174	0.4684	0.088*	
H44B	0.2084	0.3512	0.5430	0.088*	
H44C	0.2300	0.3178	0.4835	0.088*	
C45	0.2497 (4)	0.4544 (2)	0.4859 (3)	0.0578 (15)	
H45A	0.2920	0.4317	0.4828	0.087*	
H45B	0.2633	0.4616	0.5386	0.087*	
H45C	0.2400	0.4978	0.4601	0.087*	
C46	0.0759 (3)	0.3237 (2)	0.1349 (3)	0.0546 (14)	
H46A	0.0201	0.3271	0.1047	0.082*	
H46B	0.0916	0.2770	0.1364	0.082*	
H46C	0.1006	0.3516	0.1126	0.082*	
C47	−0.0779 (2)	0.48924 (18)	0.1583 (2)	0.0298 (9)	
C48	−0.1455 (2)	0.48246 (18)	0.1672 (2)	0.0281 (8)	
C49	−0.2067 (2)	0.44357 (19)	0.1142 (2)	0.0315 (9)	
H49	−0.2513	0.4387	0.1202	0.038*	
C50	−0.2050 (2)	0.41191 (19)	0.0536 (2)	0.0336 (9)	
H50	−0.2474	0.3862	0.0192	0.040*	
C51	−0.1395 (2)	0.4188 (2)	0.0443 (3)	0.0364 (10)	
H51	−0.1372	0.3979	0.0033	0.044*	
C52	−0.0778 (2)	0.45664 (19)	0.0960 (3)	0.0339 (10)	
H52	−0.0337	0.4607	0.0892	0.041*	
C53	−0.1596 (2)	0.50943 (19)	0.2295 (2)	0.0295 (9)	
C54	−0.1899 (2)	0.5741 (2)	0.2270 (2)	0.0300 (9)	
C55	−0.2122 (2)	0.5943 (2)	0.2811 (3)	0.0364 (10)	
H55	−0.2316	0.6381	0.2801	0.044*	
C56	−0.2050 (3)	0.5477 (2)	0.3363 (3)	0.0449 (12)	
H56	−0.2207	0.5605	0.3721	0.054*	
C57	−0.1756 (3)	0.4836 (2)	0.3403 (3)	0.0434 (11)	
H57	−0.1705	0.4533	0.3787	0.052*	
C58	−0.1536 (2)	0.4648 (2)	0.2865 (3)	0.0345 (10)	
C59	−0.2050 (3)	0.68606 (19)	0.1768 (3)	0.0354 (10)	
H59A	−0.1990	0.7103	0.1377	0.053*	
H59B	−0.1662	0.7016	0.2262	0.053*	
H59C	−0.2562	0.6944	0.1711	0.053*	
C60	−0.1214 (3)	0.3521 (2)	0.3364 (3)	0.0505 (13)	
H60A	−0.1026	0.3099	0.3265	0.076*	
H60B	−0.1730	0.3455	0.3305	0.076*	
H60C	−0.0870	0.3669	0.3877	0.076*	
C61	0.0064 (2)	0.59682 (19)	0.1432 (3)	0.0315 (9)	
H61	−0.0010	0.5687	0.0994	0.038*	
C62	−0.0677 (2)	0.6395 (2)	0.1133 (3)	0.0362 (10)	
H62A	−0.1119	0.6102	0.1032	0.043*	
H62B	−0.0627	0.6730	0.1515	0.043*	
C63	−0.0813 (3)	0.6754 (2)	0.0408 (3)	0.0420 (11)	
H63A	−0.1278	0.7036	0.0226	0.050*	
H63B	−0.0900	0.6417	0.0017	0.050*	
C64	−0.0122 (3)	0.7195 (2)	0.0542 (3)	0.0448 (11)	
H64A	−0.0213	0.7404	0.0063	0.054*	
H64B	−0.0066	0.7559	0.0898	0.054*	
C65	0.0622 (3)	0.6782 (2)	0.0862 (3)	0.0376 (10)	
H65A	0.1058	0.7082	0.0966	0.045*	
H65B	0.0585	0.6448	0.0484	0.045*	
C66	0.0772 (2)	0.64132 (19)	0.1594 (3)	0.0343 (10)	
H66A	0.0856	0.6744	0.1991	0.041*	
H66B	0.1236	0.6131	0.1771	0.041*	
C67	0.0060 (2)	0.58350 (19)	0.2913 (2)	0.0314 (9)	
H67	−0.0488	0.5978	0.2701	0.038*	
C68	0.0244 (3)	0.5391 (2)	0.3605 (3)	0.0365 (10)	
H68A	0.0785	0.5242	0.3830	0.044*	
H68B	−0.0086	0.4988	0.3444	0.044*	
C69	0.0115 (3)	0.5774 (2)	0.4203 (3)	0.0453 (11)	
H69A	−0.0433	0.5896	0.3993	0.054*	
H69B	0.0252	0.5481	0.4644	0.054*	
C70	0.0610 (3)	0.6411 (3)	0.4448 (3)	0.0511 (13)	
H70A	0.0497	0.6667	0.4803	0.061*	
H70B	0.1159	0.6284	0.4709	0.061*	
C71	0.0452 (3)	0.6850 (2)	0.3772 (3)	0.0515 (13)	
H71A	0.0801	0.7241	0.3944	0.062*	
H71B	−0.0080	0.7020	0.3552	0.062*	
C72	0.0558 (3)	0.6480 (2)	0.3162 (3)	0.0380 (10)	
H72A	0.1105	0.6359	0.3357	0.046*	
H72B	0.0410	0.6778	0.2724	0.046*	

### Atomic displacement parameters (Å^2^)

**Table:**

	*U*^11^	*U*^22^	*U*^33^	*U*^12^	*U*^13^	*U*^23^
Ru1	0.0215 (2)	0.01744 (15)	0.02680 (18)	0.00103 (10)	0.01469 (15)	0.00164 (10)
Cl1	0.0269 (5)	0.0244 (4)	0.0302 (5)	0.0029 (3)	0.0192 (4)	0.0034 (3)
Cl2	0.0258 (5)	0.0221 (4)	0.0314 (5)	−0.0006 (3)	0.0137 (4)	0.0002 (3)
P1	0.0193 (5)	0.0175 (4)	0.0245 (5)	0.0013 (3)	0.0122 (4)	0.0013 (3)
O1	0.0334 (17)	0.0230 (13)	0.0375 (16)	0.0036 (11)	0.0216 (14)	0.0033 (11)
O2	0.0303 (17)	0.0280 (14)	0.0454 (18)	0.0040 (12)	0.0220 (15)	0.0080 (12)
C1	0.031 (2)	0.0181 (16)	0.0247 (19)	0.0002 (15)	0.0151 (18)	0.0124 (14)
C2	0.026 (2)	0.0175 (16)	0.039 (2)	0.0127 (15)	0.0153 (19)	0.0206 (15)
C3	0.041 (3)	0.0167 (17)	0.055 (3)	0.0120 (16)	0.036 (3)	0.0093 (16)
C4	0.058 (3)	0.0095 (16)	0.036 (2)	−0.0049 (16)	0.029 (2)	−0.0083 (14)
C5	0.027 (2)	0.0149 (16)	0.033 (2)	−0.0127 (15)	0.0110 (19)	−0.0028 (14)
C6	0.029 (2)	0.0179 (16)	0.041 (2)	−0.0032 (15)	0.026 (2)	0.0066 (15)
C7	0.045 (3)	0.030 (2)	0.029 (2)	0.0005 (18)	0.020 (2)	0.0018 (16)
C8	0.057 (3)	0.029 (2)	0.036 (2)	0.0077 (19)	0.031 (2)	0.0073 (17)
C9	0.084 (5)	0.106 (5)	0.023 (3)	−0.053 (4)	0.014 (3)	−0.001 (3)
C10	0.086 (4)	0.031 (2)	0.045 (3)	−0.015 (2)	0.043 (3)	−0.0092 (19)
C11	0.023 (2)	0.0212 (17)	0.028 (2)	−0.0001 (14)	0.0136 (18)	−0.0002 (14)
C12	0.024 (2)	0.0229 (18)	0.033 (2)	0.0020 (15)	0.0155 (19)	0.0021 (15)
C13	0.019 (2)	0.042 (2)	0.038 (2)	−0.0065 (17)	0.013 (2)	−0.0056 (18)
C14	0.026 (3)	0.042 (2)	0.033 (2)	−0.0061 (18)	0.009 (2)	−0.0090 (18)
C15	0.033 (3)	0.032 (2)	0.031 (2)	−0.0037 (17)	0.015 (2)	−0.0080 (16)
C16	0.022 (2)	0.0285 (19)	0.032 (2)	0.0003 (16)	0.0147 (19)	−0.0007 (16)
C17	0.024 (2)	0.0261 (18)	0.029 (2)	−0.0012 (15)	0.0156 (18)	0.0018 (15)
C18	0.020 (2)	0.0291 (19)	0.031 (2)	−0.0022 (15)	0.0135 (18)	0.0022 (15)
C19	0.025 (2)	0.035 (2)	0.040 (2)	0.0033 (17)	0.020 (2)	0.0004 (17)
C20	0.033 (3)	0.046 (2)	0.037 (2)	−0.0030 (19)	0.024 (2)	−0.0013 (19)
C21	0.033 (3)	0.039 (2)	0.035 (2)	−0.0063 (18)	0.021 (2)	0.0060 (18)
C22	0.020 (2)	0.0284 (19)	0.034 (2)	−0.0024 (16)	0.0126 (19)	0.0013 (16)
C23	0.047 (3)	0.027 (2)	0.054 (3)	0.0074 (19)	0.032 (3)	0.0035 (19)
C24	0.037 (3)	0.031 (2)	0.058 (3)	−0.0012 (18)	0.026 (3)	0.0140 (19)
C25	0.023 (2)	0.0211 (17)	0.028 (2)	0.0020 (14)	0.0153 (18)	0.0023 (14)
C26	0.024 (2)	0.0211 (17)	0.033 (2)	0.0013 (15)	0.0141 (19)	0.0007 (14)
C27	0.034 (3)	0.0260 (19)	0.035 (2)	0.0056 (17)	0.018 (2)	0.0036 (16)
C28	0.040 (3)	0.0262 (19)	0.035 (2)	0.0031 (17)	0.021 (2)	0.0091 (16)
C29	0.035 (3)	0.0265 (19)	0.039 (2)	−0.0015 (17)	0.025 (2)	0.0033 (16)
C30	0.026 (2)	0.0222 (17)	0.029 (2)	0.0007 (15)	0.0156 (18)	0.0022 (14)
C31	0.022 (2)	0.0205 (17)	0.030 (2)	0.0032 (14)	0.0147 (18)	−0.0001 (14)
C32	0.030 (2)	0.0198 (17)	0.031 (2)	0.0030 (15)	0.0182 (19)	0.0006 (14)
C33	0.044 (3)	0.032 (2)	0.028 (2)	0.0043 (18)	0.023 (2)	0.0001 (16)
C34	0.046 (3)	0.032 (2)	0.028 (2)	0.0030 (18)	0.016 (2)	−0.0031 (16)
C35	0.043 (3)	0.0243 (19)	0.030 (2)	0.0005 (17)	0.017 (2)	−0.0013 (15)
C36	0.028 (2)	0.0201 (17)	0.031 (2)	0.0002 (15)	0.0135 (19)	0.0012 (14)
Ru2	0.0228 (2)	0.02222 (17)	0.0449 (2)	−0.00017 (11)	0.01981 (17)	−0.00099 (12)
Cl3	0.0262 (6)	0.0247 (4)	0.0429 (6)	−0.0014 (4)	0.0175 (5)	−0.0024 (4)
Cl4	0.0266 (6)	0.0308 (5)	0.0447 (6)	0.0016 (4)	0.0219 (5)	−0.0013 (4)
P2	0.0206 (6)	0.0250 (5)	0.0415 (6)	−0.0002 (4)	0.0170 (5)	−0.0031 (4)
O3	0.0325 (17)	0.0276 (14)	0.0412 (17)	0.0050 (12)	0.0221 (15)	−0.0003 (12)
O4	0.0315 (18)	0.0410 (17)	0.0512 (19)	0.0036 (13)	0.0204 (16)	0.0143 (14)
C37	0.039 (3)	0.0228 (19)	0.045 (3)	0.0032 (17)	0.024 (2)	0.0075 (17)
C38	0.023 (2)	0.0211 (18)	0.043 (2)	0.0086 (15)	0.015 (2)	0.0097 (16)
C39	0.037 (3)	0.0180 (18)	0.061 (3)	0.0073 (16)	0.031 (3)	0.0024 (18)
C40	0.051 (3)	0.0177 (19)	0.056 (3)	−0.0084 (18)	0.028 (3)	−0.0107 (18)
C41	0.032 (3)	0.0228 (19)	0.069 (3)	−0.0147 (18)	0.024 (3)	−0.002 (2)
C42	0.036 (3)	0.0231 (19)	0.069 (3)	0.0000 (17)	0.037 (3)	0.0077 (19)
C43	0.055 (3)	0.031 (2)	0.058 (3)	0.007 (2)	0.037 (3)	0.004 (2)
C44	0.086 (5)	0.039 (3)	0.062 (4)	−0.004 (3)	0.043 (3)	0.009 (2)
C45	0.088 (5)	0.044 (3)	0.043 (3)	−0.015 (3)	0.033 (3)	0.000 (2)
C46	0.067 (4)	0.035 (2)	0.060 (3)	−0.013 (2)	0.029 (3)	−0.004 (2)
C47	0.024 (2)	0.0261 (19)	0.043 (2)	−0.0002 (16)	0.019 (2)	−0.0018 (17)
C48	0.023 (2)	0.0249 (18)	0.038 (2)	0.0020 (15)	0.0152 (19)	0.0018 (16)
C49	0.022 (2)	0.035 (2)	0.041 (2)	−0.0005 (17)	0.018 (2)	0.0035 (17)
C50	0.024 (2)	0.031 (2)	0.041 (2)	−0.0032 (17)	0.012 (2)	−0.0031 (17)
C51	0.030 (3)	0.035 (2)	0.045 (3)	0.0006 (18)	0.018 (2)	−0.0094 (19)
C52	0.023 (2)	0.033 (2)	0.051 (3)	0.0003 (17)	0.022 (2)	−0.0068 (18)
C53	0.020 (2)	0.034 (2)	0.036 (2)	−0.0034 (16)	0.0142 (19)	0.0003 (17)
C54	0.022 (2)	0.038 (2)	0.033 (2)	−0.0036 (17)	0.0148 (19)	−0.0007 (17)
C55	0.026 (2)	0.045 (2)	0.043 (3)	−0.0018 (18)	0.020 (2)	−0.011 (2)
C56	0.042 (3)	0.062 (3)	0.040 (3)	−0.004 (2)	0.027 (3)	−0.006 (2)
C57	0.038 (3)	0.056 (3)	0.041 (3)	−0.009 (2)	0.023 (2)	0.000 (2)
C58	0.020 (2)	0.041 (2)	0.044 (3)	−0.0018 (17)	0.016 (2)	0.0069 (19)
C59	0.031 (3)	0.0247 (19)	0.054 (3)	0.0037 (17)	0.023 (2)	−0.0037 (18)
C60	0.041 (3)	0.048 (3)	0.055 (3)	0.001 (2)	0.017 (3)	0.022 (2)
C61	0.023 (2)	0.028 (2)	0.047 (3)	−0.0004 (16)	0.020 (2)	−0.0025 (17)
C62	0.025 (2)	0.031 (2)	0.055 (3)	0.0028 (17)	0.021 (2)	0.0000 (19)
C63	0.030 (3)	0.032 (2)	0.060 (3)	0.0019 (18)	0.018 (2)	0.005 (2)
C64	0.039 (3)	0.033 (2)	0.061 (3)	−0.001 (2)	0.022 (3)	0.007 (2)
C65	0.031 (3)	0.031 (2)	0.054 (3)	−0.0008 (18)	0.023 (2)	0.0019 (19)
C66	0.026 (2)	0.029 (2)	0.052 (3)	0.0005 (17)	0.022 (2)	−0.0025 (18)
C67	0.023 (2)	0.030 (2)	0.043 (2)	−0.0002 (16)	0.018 (2)	−0.0059 (17)
C68	0.025 (2)	0.042 (2)	0.041 (3)	0.0032 (18)	0.014 (2)	−0.0039 (19)
C69	0.034 (3)	0.060 (3)	0.046 (3)	0.002 (2)	0.022 (2)	−0.010 (2)
C70	0.034 (3)	0.070 (3)	0.054 (3)	−0.006 (2)	0.024 (3)	−0.027 (3)
C71	0.045 (3)	0.046 (3)	0.073 (4)	−0.007 (2)	0.036 (3)	−0.027 (3)
C72	0.028 (3)	0.035 (2)	0.051 (3)	−0.0016 (18)	0.018 (2)	−0.0119 (19)

### Geometric parameters (Å, º)

**Table:**

Ru1—C6	2.205 (3)	Ru2—C41	2.228 (4)
Ru1—C5	2.227 (3)	Ru2—C42	2.230 (4)
Ru1—C4	2.247 (3)	Ru2—C40	2.244 (4)
Ru1—C2	2.258 (3)	Ru2—C38	2.263 (4)
Ru1—C1	2.266 (3)	Ru2—C39	2.265 (4)
Ru1—C3	2.270 (3)	Ru2—C37	2.276 (4)
Ru1—P1	2.4206 (10)	Ru2—P2	2.4181 (11)
Ru1—Cl1	2.4441 (10)	Ru2—Cl3	2.4426 (11)
Ru1—Cl2	2.4445 (11)	Ru2—Cl4	2.4508 (11)
P1—C11	1.884 (4)	P2—C47	1.877 (4)
P1—C31	1.886 (4)	P2—C67	1.883 (4)
P1—C25	1.893 (4)	P2—C61	1.884 (4)
O1—C18	1.379 (4)	O3—C54	1.380 (5)
O1—C23	1.446 (4)	O3—C59	1.445 (4)
O2—C22	1.387 (5)	O4—C58	1.390 (5)
O2—C24	1.443 (5)	O4—C60	1.438 (5)
C1—C6	1.419 (6)	C37—C42	1.416 (7)
C1—C2	1.443 (5)	C37—C38	1.445 (6)
C1—C7	1.528 (5)	C37—C43	1.532 (7)
C2—C3	1.405 (6)	C38—C39	1.391 (6)
C2—H2	0.9500	C38—H38	0.9500
C3—C4	1.435 (7)	C39—C40	1.448 (7)
C3—H3	0.9500	C39—H39	0.9500
C4—C5	1.435 (6)	C40—C41	1.420 (6)
C4—C10	1.513 (6)	C40—C46	1.510 (7)
C5—C6	1.440 (5)	C41—C42	1.453 (7)
C5—H5	0.9500	C41—H41	0.9500
C6—H6	0.9500	C42—H42	0.9500
C7—C9	1.509 (7)	C43—C45	1.541 (7)
C7—C8	1.535 (5)	C43—C44	1.544 (6)
C7—H7	1.0000	C43—H43	1.0000
C8—H8A	0.9800	C44—H44A	0.9800
C8—H8B	0.9800	C44—H44B	0.9800
C8—H8C	0.9800	C44—H44C	0.9800
C9—H9A	0.9800	C45—H45A	0.9800
C9—H9B	0.9800	C45—H45B	0.9800
C9—H9C	0.9800	C45—H45C	0.9800
C10—H10A	0.9800	C46—H46A	0.9800
C10—H10B	0.9800	C46—H46B	0.9800
C10—H10C	0.9800	C46—H46C	0.9800
C11—C12	1.419 (5)	C47—C52	1.418 (6)
C11—C16	1.428 (5)	C47—C48	1.437 (5)
C12—C13	1.420 (6)	C48—C49	1.411 (6)
C12—C17	1.516 (5)	C48—C53	1.513 (6)
C13—C14	1.394 (6)	C49—C50	1.394 (6)
C13—H13	0.9500	C49—H49	0.9500
C14—C15	1.400 (6)	C50—C51	1.400 (6)
C14—H14	0.9500	C50—H50	0.9500
C15—C16	1.393 (6)	C51—C52	1.397 (6)
C15—H15	0.9500	C51—H51	0.9500
C16—H16	0.9500	C52—H52	0.9500
C17—C18	1.411 (5)	C53—C54	1.414 (6)
C17—C22	1.417 (5)	C53—C58	1.416 (6)
C18—C19	1.414 (5)	C54—C55	1.417 (5)
C19—C20	1.407 (6)	C55—C56	1.407 (6)
C19—H19	0.9500	C55—H55	0.9500
C20—C21	1.394 (6)	C56—C57	1.392 (7)
C20—H20	0.9500	C56—H56	0.9500
C21—C22	1.408 (5)	C57—C58	1.401 (6)
C21—H21	0.9500	C57—H57	0.9500
C23—H23A	0.9800	C59—H59A	0.9800
C23—H23B	0.9800	C59—H59B	0.9800
C23—H23C	0.9800	C59—H59C	0.9800
C24—H24A	0.9800	C60—H60A	0.9800
C24—H24B	0.9800	C60—H60B	0.9800
C24—H24C	0.9800	C60—H60C	0.9800
C25—C26	1.546 (5)	C61—C62	1.550 (6)
C25—C30	1.552 (5)	C61—C66	1.558 (5)
C25—H25	1.0000	C61—H61	1.0000
C26—C27	1.540 (5)	C62—C63	1.537 (6)
C26—H26A	0.9900	C62—H62A	0.9900
C26—H26B	0.9900	C62—H62B	0.9900
C27—C28	1.549 (6)	C63—C64	1.540 (6)
C27—H27A	0.9900	C63—H63A	0.9900
C27—H27B	0.9900	C63—H63B	0.9900
C28—C29	1.542 (6)	C64—C65	1.538 (6)
C28—H28A	0.9900	C64—H64A	0.9900
C28—H28B	0.9900	C64—H64B	0.9900
C29—C30	1.537 (5)	C65—C66	1.551 (6)
C29—H29A	0.9900	C65—H65A	0.9900
C29—H29B	0.9900	C65—H65B	0.9900
C30—H30A	0.9900	C66—H66A	0.9900
C30—H30B	0.9900	C66—H66B	0.9900
C31—C32	1.541 (5)	C67—C68	1.546 (6)
C31—C36	1.558 (5)	C67—C72	1.553 (6)
C31—H31	1.0000	C67—H67	1.0000
C32—C33	1.544 (5)	C68—C69	1.551 (6)
C32—H32A	0.9900	C68—H68A	0.9900
C32—H32B	0.9900	C68—H68B	0.9900
C33—C34	1.538 (6)	C69—C70	1.537 (7)
C33—H33A	0.9900	C69—H69A	0.9900
C33—H33B	0.9900	C69—H69B	0.9900
C34—C35	1.538 (6)	C70—C71	1.527 (8)
C34—H34A	0.9900	C70—H70A	0.9900
C34—H34B	0.9900	C70—H70B	0.9900
C35—C36	1.539 (5)	C71—C72	1.535 (6)
C35—H35A	0.9900	C71—H71A	0.9900
C35—H35B	0.9900	C71—H71B	0.9900
C36—H36A	0.9900	C72—H72A	0.9900
C36—H36B	0.9900	C72—H72B	0.9900
			
C6—Ru1—C5	37.92 (14)	C41—Ru2—C42	38.05 (18)
C6—Ru1—C4	67.73 (14)	C41—Ru2—C40	37.03 (17)
C5—Ru1—C4	37.40 (15)	C42—Ru2—C40	67.57 (16)
C6—Ru1—C2	66.40 (14)	C41—Ru2—C38	78.30 (17)
C5—Ru1—C2	78.81 (15)	C42—Ru2—C38	65.82 (15)
C4—Ru1—C2	66.52 (16)	C40—Ru2—C38	66.44 (17)
C6—Ru1—C1	36.97 (15)	C41—Ru2—C39	66.45 (17)
C5—Ru1—C1	67.67 (14)	C42—Ru2—C39	78.11 (15)
C4—Ru1—C1	79.89 (14)	C40—Ru2—C39	37.45 (18)
C2—Ru1—C1	37.20 (13)	C38—Ru2—C39	35.77 (16)
C6—Ru1—C3	78.43 (14)	C41—Ru2—C37	67.52 (18)
C5—Ru1—C3	66.58 (15)	C42—Ru2—C37	36.61 (17)
C4—Ru1—C3	37.03 (17)	C40—Ru2—C37	79.91 (17)
C2—Ru1—C3	36.15 (15)	C38—Ru2—C37	37.12 (14)
C1—Ru1—C3	66.44 (14)	C39—Ru2—C37	66.26 (15)
C6—Ru1—P1	93.14 (10)	C41—Ru2—P2	96.33 (13)
C5—Ru1—P1	96.05 (11)	C42—Ru2—P2	94.49 (11)
C4—Ru1—P1	123.63 (13)	C40—Ru2—P2	122.70 (14)
C2—Ru1—P1	152.93 (10)	C38—Ru2—P2	154.49 (11)
C1—Ru1—P1	116.23 (10)	C39—Ru2—P2	160.14 (12)
C3—Ru1—P1	160.65 (13)	C37—Ru2—P2	117.74 (11)
C6—Ru1—Cl1	147.88 (11)	C41—Ru2—Cl3	162.00 (14)
C5—Ru1—Cl1	110.06 (10)	C42—Ru2—Cl3	124.32 (13)
C4—Ru1—Cl1	85.41 (10)	C40—Ru2—Cl3	147.92 (13)
C2—Ru1—Cl1	119.60 (10)	C38—Ru2—Cl3	90.39 (11)
C1—Ru1—Cl1	156.45 (10)	C39—Ru2—Cl3	111.60 (12)
C3—Ru1—Cl1	90.82 (10)	C37—Ru2—Cl3	95.02 (12)
P1—Ru1—Cl1	87.23 (4)	P2—Ru2—Cl3	87.86 (4)
C6—Ru1—Cl2	124.08 (11)	C41—Ru2—Cl4	111.36 (14)
C5—Ru1—Cl2	161.65 (10)	C42—Ru2—Cl4	149.39 (13)
C4—Ru1—Cl2	147.67 (13)	C40—Ru2—Cl4	85.79 (13)
C2—Ru1—Cl2	89.81 (11)	C38—Ru2—Cl4	118.01 (10)
C1—Ru1—Cl2	94.58 (10)	C39—Ru2—Cl4	89.98 (12)
C3—Ru1—Cl2	111.66 (12)	C37—Ru2—Cl4	154.97 (11)
P1—Ru1—Cl2	87.53 (4)	P2—Ru2—Cl4	87.28 (4)
Cl1—Ru1—Cl2	88.04 (4)	Cl3—Ru2—Cl4	86.26 (4)
C11—P1—C31	108.60 (17)	C47—P2—C67	108.53 (18)
C11—P1—C25	96.86 (17)	C47—P2—C61	97.24 (19)
C31—P1—C25	106.60 (16)	C67—P2—C61	106.12 (18)
C11—P1—Ru1	108.18 (11)	C47—P2—Ru2	108.16 (12)
C31—P1—Ru1	119.13 (13)	C67—P2—Ru2	117.37 (14)
C25—P1—Ru1	115.06 (11)	C61—P2—Ru2	117.35 (12)
C18—O1—C23	116.7 (3)	C54—O3—C59	117.3 (3)
C22—O2—C24	117.6 (3)	C58—O4—C60	117.9 (4)
C6—C1—C2	117.3 (3)	C42—C37—C38	117.2 (4)
C6—C1—C7	120.0 (3)	C42—C37—C43	121.7 (4)
C2—C1—C7	122.2 (4)	C38—C37—C43	120.3 (4)
C6—C1—Ru1	69.20 (19)	C42—C37—Ru2	69.9 (2)
C2—C1—Ru1	71.12 (18)	C38—C37—Ru2	71.0 (2)
C7—C1—Ru1	137.1 (2)	C43—C37—Ru2	138.6 (3)
C3—C2—C1	121.5 (4)	C39—C38—C37	122.1 (4)
C3—C2—Ru1	72.4 (2)	C39—C38—Ru2	72.2 (2)
C1—C2—Ru1	71.69 (19)	C37—C38—Ru2	71.9 (2)
C3—C2—H2	119.2	C39—C38—H38	118.9
C1—C2—H2	119.2	C37—C38—H38	118.9
Ru1—C2—H2	129.1	Ru2—C38—H38	129.6
C2—C3—C4	120.9 (4)	C38—C39—C40	120.9 (4)
C2—C3—Ru1	71.5 (2)	C38—C39—Ru2	72.0 (2)
C4—C3—Ru1	70.62 (19)	C40—C39—Ru2	70.5 (2)
C2—C3—H3	119.5	C38—C39—H39	119.6
C4—C3—H3	119.5	C40—C39—H39	119.6
Ru1—C3—H3	131.3	Ru2—C39—H39	130.6
C3—C4—C5	118.7 (4)	C41—C40—C39	118.3 (4)
C3—C4—C10	120.8 (4)	C41—C40—C46	122.0 (5)
C5—C4—C10	120.5 (4)	C39—C40—C46	119.7 (4)
C3—C4—Ru1	72.3 (2)	C41—C40—Ru2	70.9 (2)
C5—C4—Ru1	70.53 (19)	C39—C40—Ru2	72.1 (2)
C10—C4—Ru1	129.9 (3)	C46—C40—Ru2	128.4 (3)
C4—C5—C6	119.3 (4)	C40—C41—C42	120.0 (4)
C4—C5—Ru1	72.1 (2)	C40—C41—Ru2	72.1 (2)
C6—C5—Ru1	70.21 (19)	C42—C41—Ru2	71.0 (2)
C4—C5—H5	120.3	C40—C41—H41	120.0
C6—C5—H5	120.3	C42—C41—H41	120.0
Ru1—C5—H5	129.8	Ru2—C41—H41	129.2
C1—C6—C5	122.1 (3)	C37—C42—C41	121.5 (4)
C1—C6—Ru1	73.83 (19)	C37—C42—Ru2	73.5 (2)
C5—C6—Ru1	71.87 (19)	C41—C42—Ru2	70.9 (2)
C1—C6—H6	119.0	C37—C42—H42	119.2
C5—C6—H6	119.0	C41—C42—H42	119.2
Ru1—C6—H6	127.5	Ru2—C42—H42	128.8
C9—C7—C1	116.4 (4)	C37—C43—C45	114.9 (4)
C9—C7—C8	110.5 (4)	C37—C43—C44	107.7 (4)
C1—C7—C8	106.9 (3)	C45—C43—C44	109.9 (4)
C9—C7—H7	107.6	C37—C43—H43	108.0
C1—C7—H7	107.6	C45—C43—H43	108.0
C8—C7—H7	107.6	C44—C43—H43	108.0
C7—C8—H8A	109.5	C43—C44—H44A	109.5
C7—C8—H8B	109.5	C43—C44—H44B	109.5
H8A—C8—H8B	109.5	H44A—C44—H44B	109.5
C7—C8—H8C	109.5	C43—C44—H44C	109.5
H8A—C8—H8C	109.5	H44A—C44—H44C	109.5
H8B—C8—H8C	109.5	H44B—C44—H44C	109.5
C7—C9—H9A	109.5	C43—C45—H45A	109.5
C7—C9—H9B	109.5	C43—C45—H45B	109.5
H9A—C9—H9B	109.5	H45A—C45—H45B	109.5
C7—C9—H9C	109.5	C43—C45—H45C	109.5
H9A—C9—H9C	109.5	H45A—C45—H45C	109.5
H9B—C9—H9C	109.5	H45B—C45—H45C	109.5
C4—C10—H10A	109.5	C40—C46—H46A	109.5
C4—C10—H10B	109.5	C40—C46—H46B	109.5
H10A—C10—H10B	109.5	H46A—C46—H46B	109.5
C4—C10—H10C	109.5	C40—C46—H46C	109.5
H10A—C10—H10C	109.5	H46A—C46—H46C	109.5
H10B—C10—H10C	109.5	H46B—C46—H46C	109.5
C12—C11—C16	118.3 (3)	C52—C47—C48	117.4 (4)
C12—C11—P1	130.7 (3)	C52—C47—P2	111.3 (3)
C16—C11—P1	110.9 (3)	C48—C47—P2	131.3 (3)
C11—C12—C13	117.7 (3)	C49—C48—C47	118.2 (4)
C11—C12—C17	128.2 (4)	C49—C48—C53	113.5 (3)
C13—C12—C17	114.1 (3)	C47—C48—C53	128.3 (4)
C14—C13—C12	123.1 (4)	C50—C49—C48	123.2 (4)
C14—C13—H13	118.5	C50—C49—H49	118.4
C12—C13—H13	118.5	C48—C49—H49	118.4
C13—C14—C15	119.0 (4)	C49—C50—C51	118.8 (4)
C13—C14—H14	120.5	C49—C50—H50	120.6
C15—C14—H14	120.5	C51—C50—H50	120.6
C16—C15—C14	119.3 (4)	C52—C51—C50	119.4 (4)
C16—C15—H15	120.4	C52—C51—H51	120.3
C14—C15—H15	120.4	C50—C51—H51	120.3
C15—C16—C11	122.4 (4)	C51—C52—C47	123.0 (4)
C15—C16—H16	118.8	C51—C52—H52	118.5
C11—C16—H16	118.8	C47—C52—H52	118.5
C18—C17—C22	117.8 (3)	C54—C53—C58	118.3 (4)
C18—C17—C12	121.7 (3)	C54—C53—C48	122.5 (4)
C22—C17—C12	119.9 (3)	C58—C53—C48	118.5 (3)
O1—C18—C17	115.1 (3)	O3—C54—C53	115.7 (3)
O1—C18—C19	123.3 (3)	O3—C54—C55	123.4 (4)
C17—C18—C19	121.6 (3)	C53—C54—C55	120.9 (4)
C20—C19—C18	118.5 (4)	C56—C55—C54	118.2 (4)
C20—C19—H19	120.8	C56—C55—H55	120.9
C18—C19—H19	120.8	C54—C55—H55	120.9
C21—C20—C19	121.5 (4)	C57—C56—C55	122.5 (4)
C21—C20—H20	119.2	C57—C56—H56	118.7
C19—C20—H20	119.2	C55—C56—H56	118.7
C20—C21—C22	119.1 (4)	C56—C57—C58	118.3 (4)
C20—C21—H21	120.5	C56—C57—H57	120.9
C22—C21—H21	120.5	C58—C57—H57	120.9
O2—C22—C21	123.6 (3)	O4—C58—C57	123.9 (4)
O2—C22—C17	115.0 (3)	O4—C58—C53	114.3 (4)
C21—C22—C17	121.5 (4)	C57—C58—C53	121.9 (4)
O1—C23—H23A	109.5	O3—C59—H59A	109.5
O1—C23—H23B	109.5	O3—C59—H59B	109.5
H23A—C23—H23B	109.5	H59A—C59—H59B	109.5
O1—C23—H23C	109.5	O3—C59—H59C	109.5
H23A—C23—H23C	109.5	H59A—C59—H59C	109.5
H23B—C23—H23C	109.5	H59B—C59—H59C	109.5
O2—C24—H24A	109.5	O4—C60—H60A	109.5
O2—C24—H24B	109.5	O4—C60—H60B	109.5
H24A—C24—H24B	109.5	H60A—C60—H60B	109.5
O2—C24—H24C	109.5	O4—C60—H60C	109.5
H24A—C24—H24C	109.5	H60A—C60—H60C	109.5
H24B—C24—H24C	109.5	H60B—C60—H60C	109.5
C26—C25—C30	111.3 (3)	C62—C61—C66	111.1 (3)
C26—C25—P1	116.1 (2)	C62—C61—P2	112.9 (3)
C30—C25—P1	116.8 (3)	C66—C61—P2	118.4 (3)
C26—C25—H25	103.5	C62—C61—H61	104.3
C30—C25—H25	103.5	C66—C61—H61	104.3
P1—C25—H25	103.5	P2—C61—H61	104.3
C27—C26—C25	108.2 (3)	C63—C62—C61	109.6 (3)
C27—C26—H26A	110.1	C63—C62—H62A	109.8
C25—C26—H26A	110.1	C61—C62—H62A	109.8
C27—C26—H26B	110.1	C63—C62—H62B	109.8
C25—C26—H26B	110.1	C61—C62—H62B	109.8
H26A—C26—H26B	108.4	H62A—C62—H62B	108.2
C26—C27—C28	111.9 (3)	C62—C63—C64	110.9 (4)
C26—C27—H27A	109.2	C62—C63—H63A	109.5
C28—C27—H27A	109.2	C64—C63—H63A	109.5
C26—C27—H27B	109.2	C62—C63—H63B	109.5
C28—C27—H27B	109.2	C64—C63—H63B	109.5
H27A—C27—H27B	107.9	H63A—C63—H63B	108.0
C29—C28—C27	113.0 (3)	C65—C64—C63	111.2 (4)
C29—C28—H28A	109.0	C65—C64—H64A	109.4
C27—C28—H28A	109.0	C63—C64—H64A	109.4
C29—C28—H28B	109.0	C65—C64—H64B	109.4
C27—C28—H28B	109.0	C63—C64—H64B	109.4
H28A—C28—H28B	107.8	H64A—C64—H64B	108.0
C30—C29—C28	111.1 (3)	C64—C65—C66	111.7 (4)
C30—C29—H29A	109.4	C64—C65—H65A	109.3
C28—C29—H29A	109.4	C66—C65—H65A	109.3
C30—C29—H29B	109.4	C64—C65—H65B	109.3
C28—C29—H29B	109.4	C66—C65—H65B	109.3
H29A—C29—H29B	108.0	H65A—C65—H65B	107.9
C29—C30—C25	108.5 (3)	C65—C66—C61	108.7 (4)
C29—C30—H30A	110.0	C65—C66—H66A	109.9
C25—C30—H30A	110.0	C61—C66—H66A	109.9
C29—C30—H30B	110.0	C65—C66—H66B	109.9
C25—C30—H30B	110.0	C61—C66—H66B	109.9
H30A—C30—H30B	108.4	H66A—C66—H66B	108.3
C32—C31—C36	109.3 (3)	C68—C67—C72	109.8 (4)
C32—C31—P1	111.4 (2)	C68—C67—P2	110.9 (3)
C36—C31—P1	113.8 (2)	C72—C67—P2	114.0 (3)
C32—C31—H31	107.3	C68—C67—H67	107.3
C36—C31—H31	107.3	C72—C67—H67	107.3
P1—C31—H31	107.3	P2—C67—H67	107.3
C31—C32—C33	112.3 (3)	C67—C68—C69	111.7 (4)
C31—C32—H32A	109.1	C67—C68—H68A	109.3
C33—C32—H32A	109.1	C69—C68—H68A	109.3
C31—C32—H32B	109.1	C67—C68—H68B	109.3
C33—C32—H32B	109.1	C69—C68—H68B	109.3
H32A—C32—H32B	107.9	H68A—C68—H68B	108.0
C34—C33—C32	110.6 (3)	C70—C69—C68	110.0 (4)
C34—C33—H33A	109.5	C70—C69—H69A	109.7
C32—C33—H33A	109.5	C68—C69—H69A	109.7
C34—C33—H33B	109.5	C70—C69—H69B	109.7
C32—C33—H33B	109.5	C68—C69—H69B	109.7
H33A—C33—H33B	108.1	H69A—C69—H69B	108.2
C35—C34—C33	110.1 (3)	C71—C70—C69	110.9 (4)
C35—C34—H34A	109.6	C71—C70—H70A	109.5
C33—C34—H34A	109.6	C69—C70—H70A	109.5
C35—C34—H34B	109.6	C71—C70—H70B	109.5
C33—C34—H34B	109.6	C69—C70—H70B	109.5
H34A—C34—H34B	108.1	H70A—C70—H70B	108.0
C34—C35—C36	112.6 (3)	C70—C71—C72	113.4 (4)
C34—C35—H35A	109.1	C70—C71—H71A	108.9
C36—C35—H35A	109.1	C72—C71—H71A	108.9
C34—C35—H35B	109.1	C70—C71—H71B	108.9
C36—C35—H35B	109.1	C72—C71—H71B	108.9
H35A—C35—H35B	107.8	H71A—C71—H71B	107.7
C35—C36—C31	110.1 (3)	C71—C72—C67	110.5 (4)
C35—C36—H36A	109.6	C71—C72—H72A	109.5
C31—C36—H36A	109.6	C67—C72—H72A	109.5
C35—C36—H36B	109.6	C71—C72—H72B	109.5
C31—C36—H36B	109.6	C67—C72—H72B	109.5
H36A—C36—H36B	108.1	H72A—C72—H72B	108.1
			
C6—C1—C2—C3	1.6 (5)	C42—C37—C38—C39	0.0 (5)
C7—C1—C2—C3	−170.8 (3)	C43—C37—C38—C39	170.0 (3)
Ru1—C1—C2—C3	54.8 (3)	Ru2—C37—C38—C39	−54.2 (3)
C6—C1—C2—Ru1	−53.2 (3)	C42—C37—C38—Ru2	54.2 (3)
C7—C1—C2—Ru1	134.4 (3)	C43—C37—C38—Ru2	−135.8 (3)
C1—C2—C3—C4	−2.0 (5)	C37—C38—C39—C40	1.2 (6)
Ru1—C2—C3—C4	52.5 (3)	Ru2—C38—C39—C40	−52.9 (3)
C1—C2—C3—Ru1	−54.5 (3)	C37—C38—C39—Ru2	54.1 (3)
C2—C3—C4—C5	2.2 (5)	C38—C39—C40—C41	−2.0 (6)
Ru1—C3—C4—C5	55.0 (3)	Ru2—C39—C40—C41	−55.6 (3)
C2—C3—C4—C10	−179.3 (3)	C38—C39—C40—C46	178.1 (4)
Ru1—C3—C4—C10	−126.4 (3)	Ru2—C39—C40—C46	124.5 (4)
C2—C3—C4—Ru1	−52.8 (3)	C38—C39—C40—Ru2	53.6 (3)
C3—C4—C5—C6	−2.1 (5)	C39—C40—C41—C42	1.7 (6)
C10—C4—C5—C6	179.4 (3)	C46—C40—C41—C42	−178.4 (4)
Ru1—C4—C5—C6	53.8 (3)	Ru2—C40—C41—C42	−54.5 (3)
C3—C4—C5—Ru1	−55.9 (3)	C39—C40—C41—Ru2	56.2 (3)
C10—C4—C5—Ru1	125.5 (3)	C46—C40—C41—Ru2	−123.9 (4)
C2—C1—C6—C5	−1.5 (5)	C38—C37—C42—C41	−0.3 (5)
C7—C1—C6—C5	171.0 (3)	C43—C37—C42—C41	−170.2 (4)
Ru1—C1—C6—C5	−55.6 (3)	Ru2—C37—C42—C41	54.4 (3)
C2—C1—C6—Ru1	54.1 (3)	C38—C37—C42—Ru2	−54.7 (3)
C7—C1—C6—Ru1	−133.3 (3)	C43—C37—C42—Ru2	135.4 (4)
C4—C5—C6—C1	1.8 (5)	C40—C41—C42—C37	−0.6 (6)
Ru1—C5—C6—C1	56.5 (3)	Ru2—C41—C42—C37	−55.6 (3)
C4—C5—C6—Ru1	−54.7 (3)	C40—C41—C42—Ru2	55.0 (3)
C6—C1—C7—C9	149.8 (4)	C42—C37—C43—C45	−148.6 (4)
C2—C1—C7—C9	−38.0 (6)	C38—C37—C43—C45	41.8 (5)
Ru1—C1—C7—C9	58.7 (6)	Ru2—C37—C43—C45	−53.4 (6)
C6—C1—C7—C8	−86.2 (4)	C42—C37—C43—C44	88.6 (5)
C2—C1—C7—C8	86.0 (4)	C38—C37—C43—C44	−81.0 (5)
Ru1—C1—C7—C8	−177.3 (3)	Ru2—C37—C43—C44	−176.3 (4)
C31—P1—C11—C12	8.6 (4)	C67—P2—C47—C52	173.9 (3)
C25—P1—C11—C12	118.7 (4)	C61—P2—C47—C52	64.2 (3)
Ru1—P1—C11—C12	−122.0 (3)	Ru2—P2—C47—C52	−57.7 (3)
C31—P1—C11—C16	−169.5 (3)	C67—P2—C47—C48	−6.9 (4)
C25—P1—C11—C16	−59.4 (3)	C61—P2—C47—C48	−116.7 (4)
Ru1—P1—C11—C16	59.8 (3)	Ru2—P2—C47—C48	121.4 (4)
C16—C11—C12—C13	4.5 (5)	C52—C47—C48—C49	0.5 (6)
P1—C11—C12—C13	−173.6 (3)	P2—C47—C48—C49	−178.5 (3)
C16—C11—C12—C17	−173.0 (3)	C52—C47—C48—C53	177.4 (4)
P1—C11—C12—C17	9.0 (6)	P2—C47—C48—C53	−1.7 (6)
C11—C12—C13—C14	−2.1 (6)	C47—C48—C49—C50	−0.4 (6)
C17—C12—C13—C14	175.7 (4)	C53—C48—C49—C50	−177.8 (4)
C12—C13—C14—C15	−1.1 (6)	C48—C49—C50—C51	0.0 (6)
C13—C14—C15—C16	1.8 (6)	C49—C50—C51—C52	0.3 (6)
C14—C15—C16—C11	0.7 (6)	C50—C51—C52—C47	−0.2 (7)
C12—C11—C16—C15	−3.9 (5)	C48—C47—C52—C51	−0.2 (6)
P1—C11—C16—C15	174.5 (3)	P2—C47—C52—C51	179.0 (3)
C11—C12—C17—C18	−95.8 (5)	C49—C48—C53—C54	−94.7 (5)
C13—C12—C17—C18	86.7 (5)	C47—C48—C53—C54	88.3 (5)
C11—C12—C17—C22	92.6 (5)	C49—C48—C53—C58	75.2 (5)
C13—C12—C17—C22	−85.0 (5)	C47—C48—C53—C58	−101.8 (5)
C23—O1—C18—C17	167.6 (4)	C59—O3—C54—C53	−163.0 (3)
C23—O1—C18—C19	−11.5 (5)	C59—O3—C54—C55	16.5 (6)
C22—C17—C18—O1	−179.3 (3)	C58—C53—C54—O3	−179.2 (4)
C12—C17—C18—O1	8.9 (5)	C48—C53—C54—O3	−9.2 (6)
C22—C17—C18—C19	−0.2 (6)	C58—C53—C54—C55	1.3 (6)
C12—C17—C18—C19	−172.0 (4)	C48—C53—C54—C55	171.2 (4)
O1—C18—C19—C20	179.3 (4)	O3—C54—C55—C56	179.2 (4)
C17—C18—C19—C20	0.2 (6)	C53—C54—C55—C56	−1.4 (6)
C18—C19—C20—C21	0.2 (6)	C54—C55—C56—C57	1.2 (7)
C19—C20—C21—C22	−0.6 (7)	C55—C56—C57—C58	−1.0 (7)
C24—O2—C22—C21	2.0 (6)	C60—O4—C58—C57	5.3 (6)
C24—O2—C22—C17	−178.8 (4)	C60—O4—C58—C53	−175.3 (4)
C20—C21—C22—O2	179.8 (4)	C56—C57—C58—O4	−179.7 (4)
C20—C21—C22—C17	0.7 (6)	C56—C57—C58—C53	1.0 (7)
C18—C17—C22—O2	−179.5 (3)	C54—C53—C58—O4	179.5 (4)
C12—C17—C22—O2	−7.5 (5)	C48—C53—C58—O4	9.1 (5)
C18—C17—C22—C21	−0.3 (6)	C54—C53—C58—C57	−1.1 (6)
C12—C17—C22—C21	171.7 (4)	C48—C53—C58—C57	−171.4 (4)
C11—P1—C25—C26	−53.1 (3)	C47—P2—C61—C62	57.1 (3)
C31—P1—C25—C26	58.7 (3)	C67—P2—C61—C62	−54.7 (3)
Ru1—P1—C25—C26	−166.9 (2)	Ru2—P2—C61—C62	171.8 (2)
C11—P1—C25—C30	172.4 (3)	C47—P2—C61—C66	−170.7 (3)
C31—P1—C25—C30	−75.9 (3)	C67—P2—C61—C66	77.6 (3)
Ru1—P1—C25—C30	58.6 (3)	Ru2—P2—C61—C66	−55.9 (3)
C30—C25—C26—C27	−61.2 (4)	C66—C61—C62—C63	59.2 (5)
P1—C25—C26—C27	161.8 (3)	P2—C61—C62—C63	−165.2 (3)
C25—C26—C27—C28	55.3 (4)	C61—C62—C63—C64	−57.5 (5)
C26—C27—C28—C29	−52.5 (4)	C62—C63—C64—C65	56.4 (5)
C27—C28—C29—C30	52.8 (4)	C63—C64—C65—C66	−56.2 (5)
C28—C29—C30—C25	−56.3 (4)	C64—C65—C66—C61	56.3 (4)
C26—C25—C30—C29	62.2 (4)	C62—C61—C66—C65	−58.0 (4)
P1—C25—C30—C29	−161.3 (2)	P2—C61—C66—C65	169.0 (3)
C11—P1—C31—C32	−92.0 (3)	C47—P2—C67—C68	85.1 (3)
C25—P1—C31—C32	164.6 (3)	C61—P2—C67—C68	−171.3 (3)
Ru1—P1—C31—C32	32.3 (3)	Ru2—P2—C67—C68	−37.9 (3)
C11—P1—C31—C36	143.8 (3)	C47—P2—C67—C72	−150.4 (3)
C25—P1—C31—C36	40.4 (3)	C61—P2—C67—C72	−46.8 (4)
Ru1—P1—C31—C36	−91.8 (3)	Ru2—P2—C67—C72	86.7 (3)
C36—C31—C32—C33	−57.2 (4)	C72—C67—C68—C69	57.4 (5)
P1—C31—C32—C33	176.2 (3)	P2—C67—C68—C69	−175.7 (3)
C31—C32—C33—C34	57.2 (5)	C67—C68—C69—C70	−57.7 (5)
C32—C33—C34—C35	−55.0 (5)	C68—C69—C70—C71	55.3 (5)
C33—C34—C35—C36	56.3 (5)	C69—C70—C71—C72	−55.2 (5)
C34—C35—C36—C31	−57.0 (5)	C70—C71—C72—C67	54.9 (5)
C32—C31—C36—C35	56.0 (4)	C68—C67—C72—C71	−54.8 (5)
P1—C31—C36—C35	−178.7 (3)	P2—C67—C72—C71	−179.9 (3)

### Hydrogen-bond geometry (Å, º)

Cg3 and Cg8 are the centroids of rings C17–C22 and C53–C58, respectively.

**Table:**

*D*—H···*A*	*D*—H	H···*A*	*D*···*A*	*D*—H···*A*
C6—H6···O2	0.95	2.50	3.328 (6)	146
C30—H30*A*···Cl2	0.99	2.74	3.552 (4)	139
C45—H45*C*···Cl3	0.98	2.79	3.577 (5)	137
C46—H46*C*···Cl4	0.98	2.68	3.302 (4)	122
C62—H62*A*···O3	0.99	2.58	3.261 (6)	126
C66—H66*B*···Cl4	0.99	2.71	3.388 (4)	126
C72—H72*A*···Cl3	0.99	2.78	3.617 (6)	143
C38—H38···Cl1	0.95	2.70	3.376 (4)	129
C33—H33*B*···*Cg*3	0.99	2.97	3.703 (5)	132
C69—H69*A*···*Cg*8	0.99	2.91	3.649 (6)	132
C60—H60*B*···*Cg*3^i^	0.98	2.84	3.655 (6)	142
C24—H24*B*···*Cg*8^ii^	0.98	2.85	3.710 (6)	147

Symmetry codes: (i) *x*−1, *y*, *z*; (ii) *x*+1, *y*, *z*.
